# Supplementary material for: Toll-like receptor 4 and macrophage scavenger receptor 1 crosstalk regulates phagocytosis of a fungal pathogen
Source: Nat Commun. 2023 Aug 14;14:4895. doi: 10.1038/s41467-023-40635-w (PMC10425417; doi:10.1038/s41467-023-40635-w)
Supplement: Supplementary file 1 — Supplementary Information [file 41467_2023_40635_MOESM1_ESM.pdf]

Supplementary Information

Toll-Like Receptor 4 and Macrophage Scavenger Receptor 1 crosstalk regulates phagocytosis of a fungal pathogen.

Chinaemerem U. Onyishi[1], Guillaume E. Desanti[1], Alex L. Wilkinson[1], Samuel Lara-Reyna[1], Eva-Maria Frickel[1], Gyorgy Fejer[2], Olivier D. Christophe[3], Clare E. Bryant[4], Subhankar Mukhopadhyay[5], Siamon Gordon[6,7], Robin C. May*[1]

Affiliations

[1] Institute of Microbiology & Infection and School of Biosciences, University of Birmingham, Edgbaston, Birmingham, B15 2TT, United Kingdom

[2] School of Biomedical Sciences, Faculty of Health, University of Plymouth, Plymouth, United Kingdom

[3] Université Paris-Saclay, INSERM, Hémostase inflammation thrombose HITH U1176, 94276, Le Kremlin-Bicêtre, France

[4] University of Cambridge, Department of Medicine, Box 157, Level 5, Addenbrooke's Hospital, Hills Road, Cambridge, CB2 0QQ, United Kingdom

[5] Peter Gorer Department of Immunobiology, School of Immunology & Microbial Sciences, King's College London, SE1 9RT, United Kingdom

[6] Department of Microbiology and Immunology, College of Medicine, Chang Gung University, Taoyuan, Taiwan

[7] Sir William Dunn School of Pathology, University of Oxford, Oxford, UK

* r.c.may@bham.ac.uk

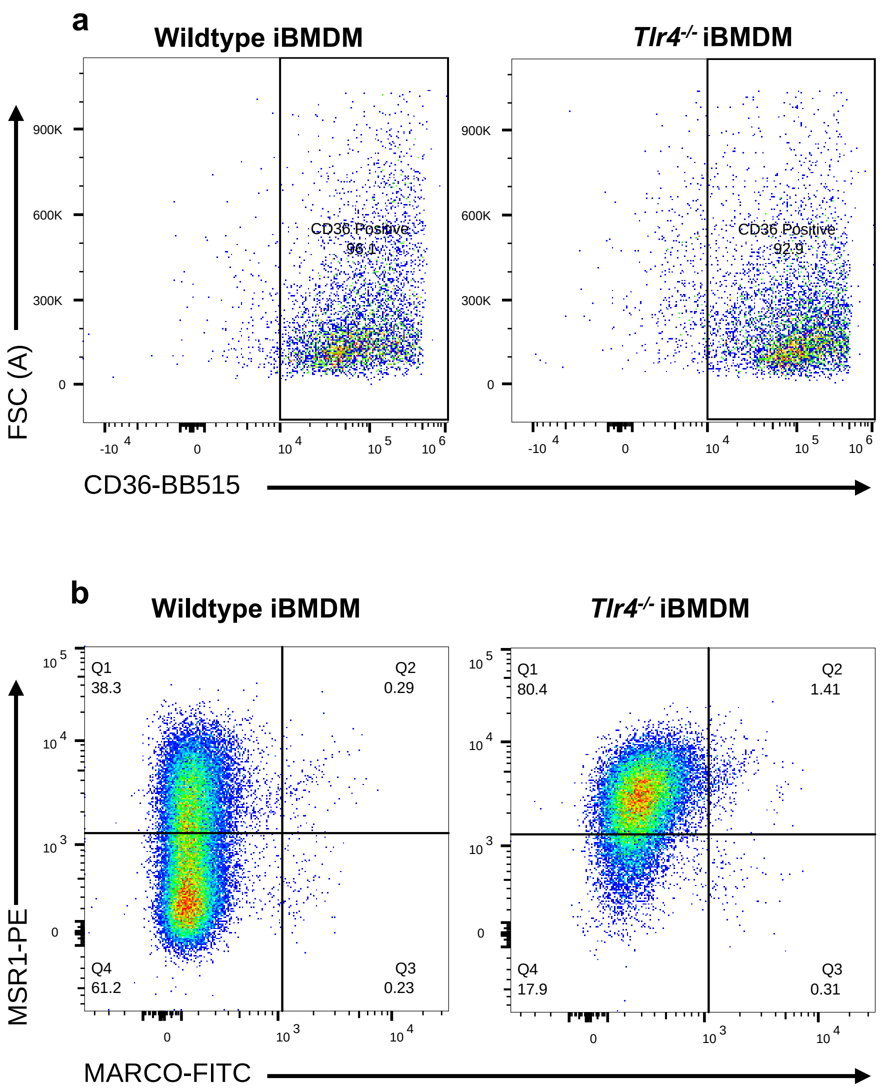

Supplementary Figure 1: Pseudocolor plots of CD36, MARCO and MSR1 expression in wildtype and *Tlr4*$^{-/-}$ macrophages. (a) Cell surface expression of CD36 was measured using anti-mouse CD35-BB515 antibody. (b) Macrophages were stained with anti-mouse MSR1 (CD204)-PE antibody and anti-MARCO-FITC antibody. Receptor expression was measured using flow cytometry and analysed using the FlowJo software. A rectangular gate (a) or quadrant gate (b) was applied to show proportion of CD36-positive cells or MSR1- and MARCO-positive cells. Data is representative of three independent experiments.

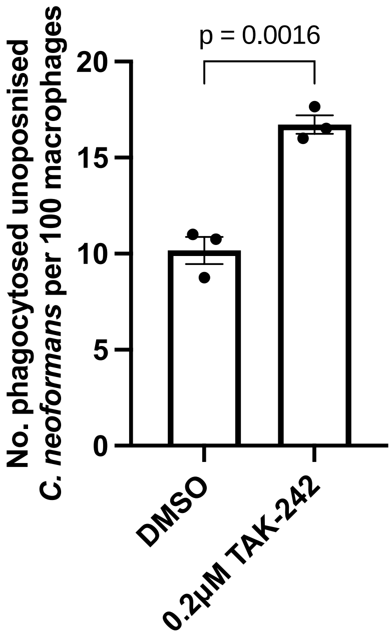

Supplementary Figure 2: Wildtype MPI cells show increased uptake following TAK-242 treatment. Wildtype MPI cells were treated with DMSO (control) or 0.2 µM TAK-242, a TLR4 specific inhibitor, for 1 h before infection with non-opsonised *C. neoformans* (*n* = 3 per condition). Phagocytosis was quantified as the number of internalised cryptococci within 100 macrophages. Data is representative of three independent experiments. Data is mean ± SEM and analysed using an unpaired two-sided t-test; P-value is shown above the graph. Source data are provided as a Source Data file.

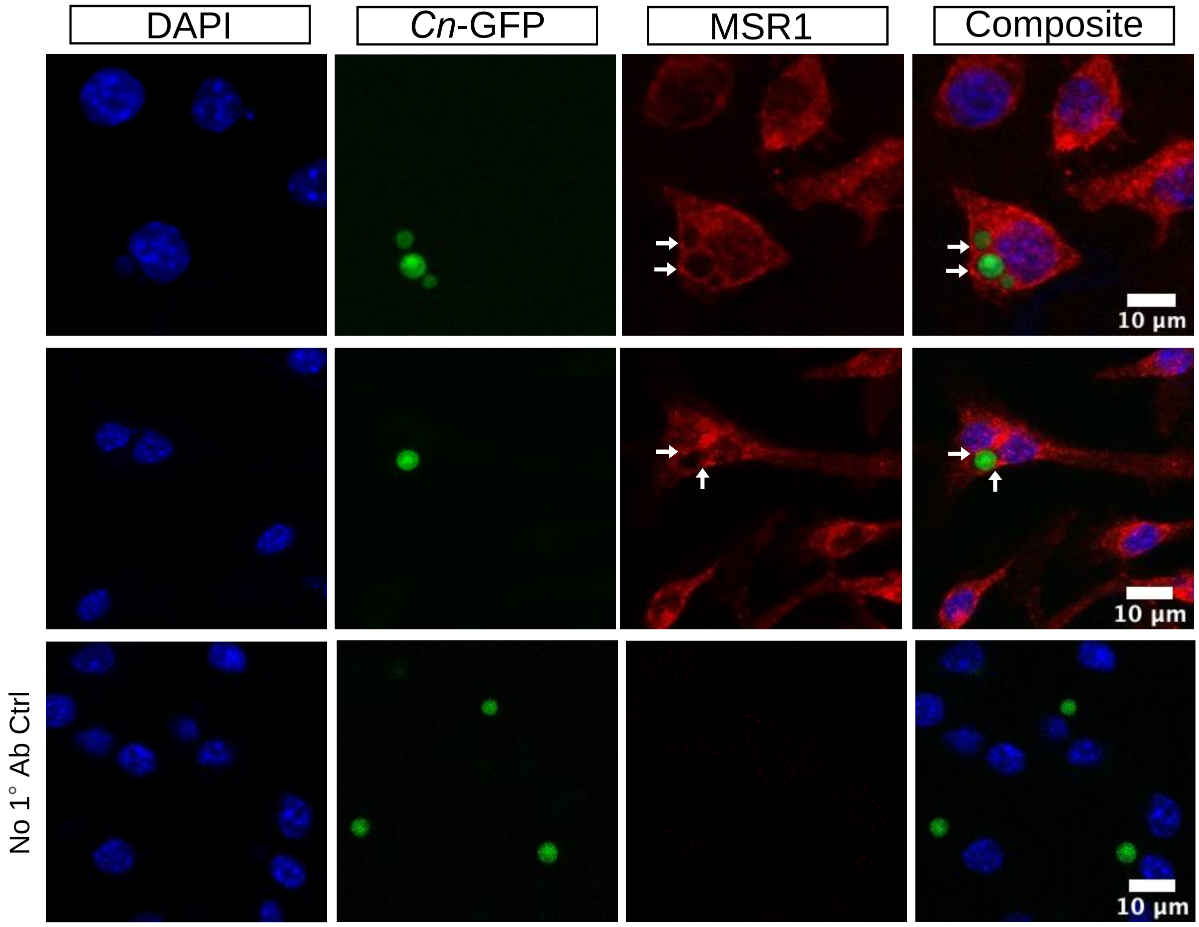

Supplementary Figure 3: Immunofluorescence analysis of Macrophage Scavenger Receptor 1 (MSR1) localisation on *Tlr4*$^{-/-}$ macrophages after infection with GFP-expressing *C. neoformans* (*Cn*-GFP). Post infection, macrophages were fixed, permeabilised and stained with rabbit anti-mouse MSR1 followed by Alexa Fluor 594 conjugated anti-rabbit IgG F(ab')$_2$ fragment secondary antibody. VECTASHIELD hard-set antifade mounting medium containing DAPI was used to stain the nucleus. Samples were captured on the Zeiss LSM880 confocal microscope using 63X Oil magnification. Representative image shows MSR1 accumulation around phagocytosed *C. neoformans* (white arrows). 'No 1° Ab Ctrl' panel shows macrophages that were not exposed to MSR1 primary antibody but exposed to Alexa Fluor 594 secondary antibody. Figure is representative of three independent experiments. Scale bar = 10 µm

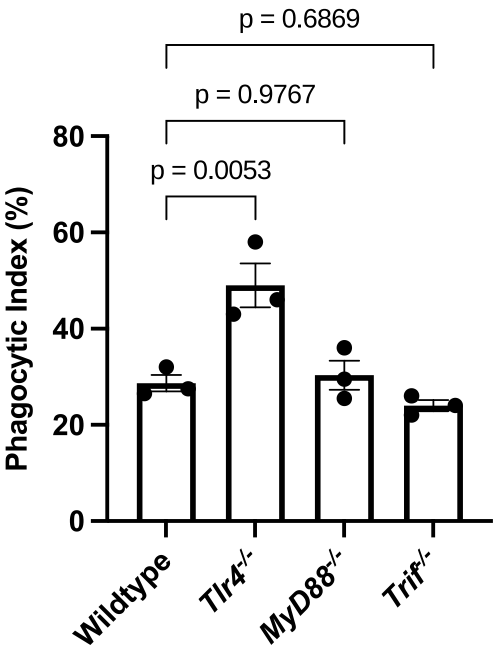

Supplementary Figure 4: *Candida albicans* infection of immortalised bone marrow derived macrophages (iBMDMs). iBMDMs isolated from wildtype, *Tlr4*$^{-/-}$, *MyD88*$^{-/-}$ and *Trif*$^{-/-}$ mice were infected with CAF2-1 *Candida albicans* expressing dTomato fluorescent protein (Caf2-dTomato) at a multiplicity of infection of 5 *C. albicans* to 1 macrophage for 45 mins (*n* = 3 per condition). Phagocytosis was quantified as the percentage of macrophages that internalised at least one fungal cell, termed phagocytic index. Data is representative of two independent experiments. Data shown as mean ± SEM; statistical significance was tested using a one-way analysis of variance (ANOVA) followed by Tukey's post-hoc test. P-values are shown above the graph. Source data are provided as a Source Data file.

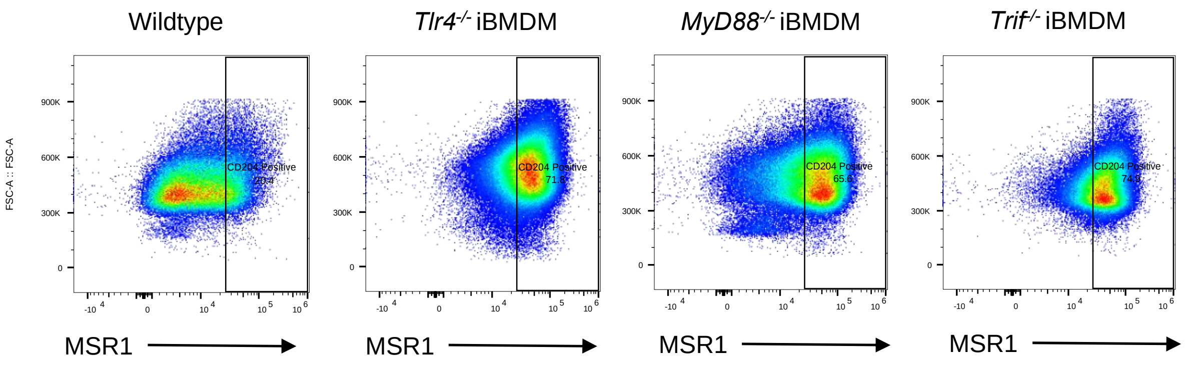

Supplementary Figure 5: MSR1 expression in wildtype, *Tlr4⁻/⁻*, *MyD88⁻/⁻* and *Trif⁻/⁻* immortalised bone marrow derived macrophages (iBMDMs). Macrophages were stained with anti-mouse CD204-PE antibody to measure the cell surface expression of MSR1. Receptor expression was measured using flow cytometry and analysed using the FlowJo software. PE-labelled rat IgG2a κ was used as an isotype control. FMO controls were used to set gates. Data is representative of two independent experiments. The proportion of MSR1 positive cells was 20.4% for wildtype, 71.8% for *Tlr4⁻/⁻*, 65.6% for *MyD88⁻/⁻* and 74.9% for *Trif⁻/⁻* macrophages.

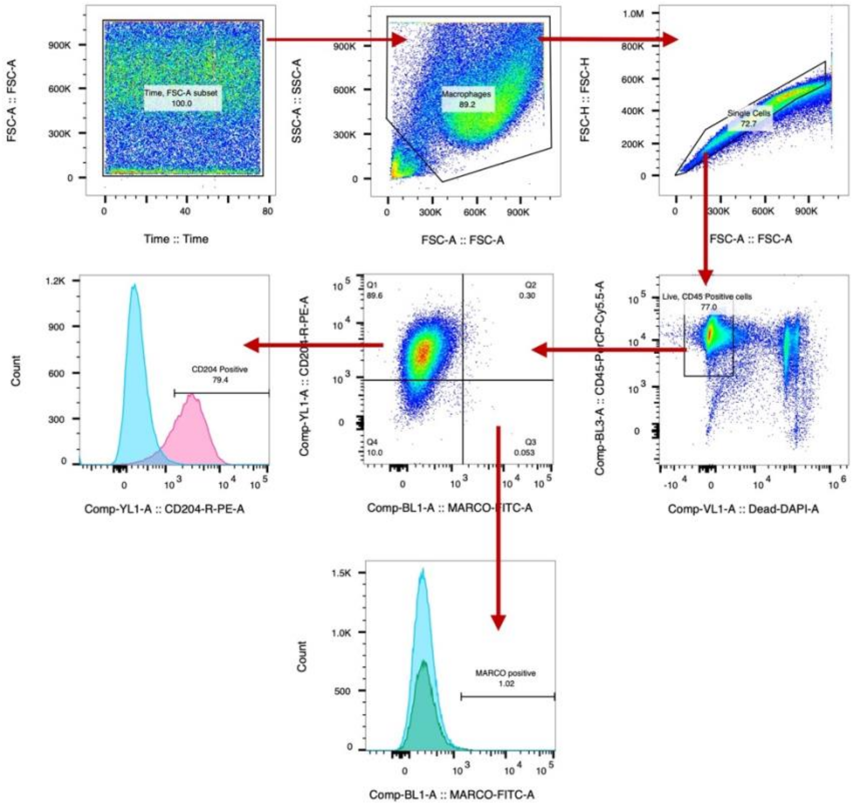

Supplementary Figure 6: Flow cytometry gating strategy. The above example shows *Tlr4*$^{-/-}$ macrophages stained with anti-CD204, PE conjugated and anti-MARCO, FITC conjugated antibodies. Following macrophage staining with relevant antibodies, cells were run through the Attune NxT flow cytometer and data acquired using the Attune NxT software. Data analysis was performed using FlowJo v10 software. Firstly, a time gate was applied to examine the quality of the sample run. Next, gates were applied to exclude debris and doublets. Anti-CD45-PerCP-Cyanine5.5 was used to identify total leukocytes, and DAPI was used to exclude dead cells. From the resulting population of live CD45+ cells, gates were applied to detect CD204 (MSR1) and MARCO scavenger receptor positive populations.
