# Supplementary material for: Toll-like receptor 4 and macrophage scavenger receptor 1 crosstalk regulates phagocytosis of a fungal pathogen
Source: Nat Commun. 2023 Aug 14;14:4895. doi: 10.1038/s41467-023-40635-w (PMC10425417; doi:10.1038/s41467-023-40635-w)
Supplement: Supplementary file 3 — Description of Additional Supplementary Files [file 41467_2023_40635_MOESM3_ESM.pdf]

Description of Additional Supplementary Files

File Name: Supplementary Data 1
Description: List of inhibitors and ligands used to block macrophage pattern recognition receptors (PRRs) and their downstream signalling molecules.
