# Supplementary material for: Toll-like receptor 4 and macrophage scavenger receptor 1 crosstalk regulates phagocytosis of a fungal pathogen
Source: Nat Commun. 2023 Aug 14;14:4895. doi: 10.1038/s41467-023-40635-w (PMC10425417; doi:10.1038/s41467-023-40635-w)
Supplement: Supplementary file 5 — Reporting Summary [file 41467_2023_40635_MOESM5_ESM.pdf]

nature portfolio

Corresponding author(s): Robin C. May

Last updated by author(s): Jul 24, 2023

Reporting Summary

Nature Portfolio wishes to improve the reproducibility of the work that we publish. This form provides structure for consistency and transparency in reporting. For further information on Nature Portfolio policies, see our Editorial Policies and the Editorial Policy Checklist.

Statistics

For all statistical analyses, confirm that the following items are present in the figure legend, table legend, main text, or Methods section.

n/a	Confirmed	
☐	☒	The exact sample size (n) for each experimental group/condition, given as a discrete number and unit of measurement
☐	☒	A statement on whether measurements were taken from distinct samples or whether the same sample was measured repeatedly
☐	☒	The statistical test(s) used AND whether they are one- or two-sided<br>*Only common tests should be described solely by name; describe more complex techniques in the Methods section.*
☒	☐	A description of all covariates tested
☐	☒	A description of any assumptions or corrections, such as tests of normality and adjustment for multiple comparisons
☐	☒	A full description of the statistical parameters including central tendency (e.g. means) or other basic estimates (e.g. regression coefficient) AND variation (e.g. standard deviation) or associated estimates of uncertainty (e.g. confidence intervals)
☐	☒	For null hypothesis testing, the test statistic (e.g. F, t, r) with confidence intervals, effect sizes, degrees of freedom and P value noted<br>*Give P values as exact values whenever suitable.*
☒	☐	For Bayesian analysis, information on the choice of priors and Markov chain Monte Carlo settings
☒	☐	For hierarchical and complex designs, identification of the appropriate level for tests and full reporting of outcomes
☒	☐	Estimates of effect sizes (e.g. Cohen's d, Pearson's r), indicating how they were calculated

Our web collection on statistics for biologists contains articles on many of the points above.

Software and code

Policy information about availability of computer code

Data collection	Phagocytosis was quantified from images from a fluorescence microscope. Images were acquired on the Zeiss Axio Observer Inverted Microscope using ZEN Blue v3.1 software. Confocal microscopy images were acquired on the Zeiss LSM880 Confocal microscope using the Zen Black v3.0 software. For flow cytometry, samples were run on the Attune NxT flow cytometer and accompanying Attune NxT software v3.1.2.
Data analysis	Numerical data were graphed and inferential statistics performed using GraphPad Prism version 9.5.0 Software. Fluorescent microscopy images were analysed using the Fiji software for Mac OS version 2.0.0. Flow cytometry data was analysed in FlowJo ver 10.8.1 for Mac OS.

For manuscripts utilizing custom algorithms or software that are central to the research but not yet described in published literature, software must be made available to editors and reviewers. We strongly encourage code deposition in a community repository (e.g. GitHub). See the Nature Portfolio guidelines for submitting code & software for further information.

Data

Policy information about availability of data

All manuscripts must include a data availability statement. This statement should provide the following information, where applicable:

- Accession codes, unique identifiers, or web links for publicly available datasets
- A description of any restrictions on data availability
- For clinical datasets or third party data, please ensure that the statement adheres to our policy

Source data are provided with this paper

Research involving human participants, their data, or biological material

Policy information about studies with human participants or human data. See also policy information about sex, gender (identity/presentation), and sexual orientation and race, ethnicity and racism.

Reporting on sex and gender	Human leukocyte cones were collected from the National Health Service Blood Transfusion service and no information on sex and gender were provided.
Reporting on race, ethnicity, or other socially relevant groupings	Information on race, ethnicity or other socially relevant groups were not provided by the National Health Service Blood Transfusion service.
Population characteristics	Leukocyte cones were provided by the NHS blood transfusion center at the Queen Elizabeth Hospital Birmingham, England. Blood donors to the NHS blood transfusion service are generally fit and well, aged between 17 to 65 years old and weigh between 50kg to 158kg. No further population characteristics were provided by the blood transfusion center. Details of individual donors are kept confidential. This was not an issue since inter-donor comparisons were not relevant in this study.
Recruitment	No human research participants were directly recruited for this study. Though human blood samples were used and they came from healthy volunteers to an NHS blood transfusion center at the Queen Elizabeth Hospital Birmingham, England.
Ethics oversight	Leukocyte cones used for this study were obtained with ethical approval from the Science, Technology, Engineering and Mathematics Ethical Review Committee at the University of Birmingham (ERN15_0804c). Informed consent was obtained prior to blood donation. All samples were anonymised and destroyed after experimentation.

Note that full information on the approval of the study protocol must also be provided in the manuscript.

Field-specific reporting

Please select the one below that is the best fit for your research. If you are not sure, read the appropriate sections before making your selection.

☒ Life sciences ☐ Behavioural & social sciences ☐ Ecological, evolutionary & environmental sciences

For a reference copy of the document with all sections, see nature.com/documents/nr-reporting-summary-flat.pdf

Life sciences study design

All studies must disclose on these points even when the disclosure is negative.

Sample size	Experiments were typically conducted in technical triplicate on three independent biological samples, except where otherwise noted. Previous work from several groups, including our own (PMID: 29643192; PMID: 36005449; PMID: 15155193; PMID: 17283107), has shown this sample size to provide a robust balance between statistical power and experimental feasibility.
Data exclusions	No data were excluded from this study.
Replication	All results in the manuscript were replicated at least twice in independent experiments. All replications gave similar results.
Randomization	Images were captured from four or more random fields in each well. After image acquisition, the fields were assigned a number and Google's random number generator was used to select the fields to be counted.
Blinding	Timelapse movies were partially blinded, since samples cannot be visually distinguished during scoring.

Reporting for specific materials, systems and methods

We require information from authors about some types of materials, experimental systems and methods used in many studies. Here, indicate whether each material, system or method listed is relevant to your study. If you are not sure if a list item applies to your research, read the appropriate section before selecting a response.

Materials & experimental systems

n/a	Involved in the study	
	☒	Antibodies
	☒	Eukaryotic cell lines
☒		Palaeontology and archaeology
☒		Animals and other organisms
☒		Clinical data
☒		Dual use research of concern
☒		Plants

Methods

n/a	Involved in the study	
☒		ChIP-seq
	☒	Flow cytometry
☒		MRI-based neuroimaging

Antibodies

Antibodies used

Flow Cytometry: rat anti-mouse CD16/CD32 Fc block 2.5ug/mL [BD Biosciences; Cat#: 553142; Clone 2.4G2]; CD45-PerCP-Cyanine5.5 0.5ug/mL [ThermoFisher; Cat#: 45-0451-82; Clone 30-F11]; anti-mouse CD204 (MSR1)-PE 0.25 ug/mL [Fisher Scientific; Cat#: 12-204-682; Clone M204PA], anti-mouse CD36-BB515 0.25 ug/mL [BD Biosciences; Cat#: 565933; Clone CRF D-2712]; anti-mouse MARCO-Fluorescein 10uL/100uL[Biotechne; Cat#: FAB2956F; Clone # 579511]; PE rat IgG2a, isotype control 0.25 ug/mL [Fisher Scientific; Cat#: 15248769; Clone eBR2a], BB515 Mouse IgA, isotype control 0.25 ug/mL [BD Biosciences; Cat#: 565095; Clone M18-254], and Rat IgG1 Fluorescein isotype control 10uL/100uL [Biotechne; Cat#: IC005F; Clone # 43414].

Confocal Microscopy: anti-mouse MSR1 1:100 [Cell Signaling Technology; Cat#: 91119; Clone: E4H1C]; Alexa Fluor 594 conjugated anti-rabbit IgG F(ab')2 fragment secondary antibody 1:500 [Cell Signaling Technology; Cat#: 8889S].

Blocking antibody: rat anti-mouse CD16/CD32 Fc block 1.25 -2.5 ug/mL [BD Biosciences; Cat#: 553142; Clone 2.4G2]; PE-Rat IgG2b isotype control 1.25 ug/mL [Invitrogen; Cat#: 15228759; Clone: eB149/10H5]

Opsonisation: anti-glucuronoxylomannan (GXM) 10ug/mL [gift from Arturo Casadevall; Clone 18B7]. Now also commercially available through Sigma Cat #MABF2069.

Validation

Primary antibodies were validated by the manufacturers:
1. anti-mouse CD16/CD32 Fc block is reactive to Mouse. Applicable in flow cytometry, blocking, immunohistochemistry and immunoprecipitation.
2. anti-CD45 erCP-Cy5.5 was validated using flow cytometric analysis of mouse bone marrow cells and splenocytes.
3. anti-CD204-PE binds mouse CD204 and does not cross react with human or rat CD204. It has been tested by flow cytometry analysis of mouse resident peritoneal cells.
4. anti-CD36 BB515 specifically binds to mouse and rat CD36 and was validated using flow cyometry analysis of mouse bone marrow cells.
5. anti-MARCO FITC detects mouse MARCO in direct ELISAs. Also detects MARCO in J774 murine macrophages, mouse dendritic cell and mouse splenocytes via flow cytometry.
6. anti-mouse MSR1 clone E4H1C is reactive to mouse with no evidence of cross-reactivity. Antibody was validated in confocal immunofluorescence analysis of fixed mouse organ sections and on RAW264.7 murine macrophage cell line. Antibody was also used in flow cytometry analysis.
7. anti-GXM 18B7 antibody detects cryptococcus GXM in ELISA, immunfluorescence,immunocytochemstry and immunodepletion analysis.

Necessary controls were included in our experiments to investigate non-specific binding:
1. Flow Cytometry: Fluorescent minus one controls and isotype controls were used to validate the specificity of antibody binding and set appropriate gates. CD36, MARCO, and MSR1 (CD204) antibodies were titrated before use to determine the appropriate concentrations.
2. Confocal Microscopy: specificity of the secondary antibody was tested by including a negative control sample that was not exposed to a primary antibody, but was exposed to the secondary antibody. A representative image of the 'No primary antibody control' sample is given in supplementary figure 3.

Eukaryotic cell lines

Policy information about cell lines and Sex and Gender in Research

Cell line source(s)

J774A.1 cells, isolated from adult, female mouse with reticulum cell sarcoma, were purchased from ATCC. Wild-type, Tlr4-/-, Myd88-/- and Trif-/- immortalized bone marrow-derived macrophages were a gift from Professor Clare Bryant. These cells were initially isolated from 8-12 week old C57BL/6 mice. Wild type, Msr1-/-, Marco-/- and Msr1/Marco double knockout MPI macrophages were a gift from Dr Subhankar Mukhopadhyay, Professor Siamon Gordon and Dr Gyorgy Fejer. MPI cells were generated from fetal liver of 15 to 19 day old C57BL/6 mice.

Authentication

J774 cells were authenticated by the manufacturers using short tandem repeat profiling. Wildtype, Tlr4-/-, MyD88-/- and Trif-/- iBMDMs were authenticated by performing an IL12p40 ELISA after LPS stimulation. They were also validated by PCR using gene specific primers. MPI cells were validated as macrophages by measuring the expression of myeloid cell-specific surface markers and their global gene expression profile. Knockout MPI cells were authenticated using immunofluorescence staining and qPCR.

Mycoplasma contamination

All cell lines were negative for mycoplasma contamination

Commonly misidentified lines (See ICLAC register)	No commonly misidentified cell lines were used in this study

Flow Cytometry

Plots

Confirm that:

☒ The axis labels state the marker and fluorochrome used (e.g. CD4-FITC).

☒ The axis scales are clearly visible. Include numbers along axes only for bottom left plot of group (a 'group' is an analysis of identical markers).

☒ All plots are contour plots with outliers or pseudocolor plots.

☒ A numerical value for number of cells or percentage (with statistics) is provided.

Methodology

Sample preparation	Macrophage cell lines seeded onto 24-well plates were lifted using TrypLE Express Enzyme. Cell suspension were incubated with rat anti-mouse CD16/CD32 Fc block diluted in FACS buffer (1XPBS without Mg2+ and Ca2+ supplemented with 2% heat inactivated FBS and 2 mM EDTA) for 10mins at 4C, then stained with appropriate antibodies for 10mins at 4C. Samples were washed in FACS buffer. DAPI (1:10,000) was used to stain dead cells.
Instrument	Attune NxT Flow Cytometer
Software	Flow data was collected using Attune NxT Flow cytometry software; Flow data was analysed using FlowJo 10.8.1 for Mac OS
Cell population abundance	Flow cytometry was performed on macrophage cell lines. Hence aside from debris and dead cells 70% to 80% of the sample population were live CD45 positive cells.
Gating strategy	Firstly, a time gate was applied to check the quality and consistency of the flow run. Debris were excluded, then gating for single cells was determined using FCS(A) on the x-axis and FSC(H) on the y-axis. Viable cells were DAPI negative and macrophages were detected using anti-CD45 antibody.

☒ Tick this box to confirm that a figure exemplifying the gating strategy is provided in the Supplementary Information.
